# Linking Costs and Quality in Healthcare: Towards Sustainable Healthcare Systems: Comment on "Hospitals Bending the Cost Curve With Increased Quality: A Scoping Review Into Integrated Hospital Strategies"

**DOI:** 10.34172/ijhpm.2022.7461

**Published:** 2022-07-30

**Authors:** Roland Bal, Iris Wallenburg

**Affiliations:** Erasmus School of Health Policy and Management, Erasmus University Rotterdam, Rotterdam, The Netherlands.

**Keywords:** Quality Improvement, Cost Containment, Complex Interventions

## Abstract

Organisation-wide studies in cost and quality of care are rare, and Wackers et al make a valuable contribution in synthesizing the literature on this issue. Their paper provides a good overview of initiatives and a list of factors that help in furthering organisation-wide change. The eleven factors they distill from the literate however remain rather abstract and more work needs to be done to contextualize the factors and the work that is needed to accomplish them and to see how they are aligned. Challenges in healthcare quality and costs moreover increasingly cross organizational boundaries and we need new methods to study and evaluate these.

 Healthcare leaders face the tremendous challenge of burdened healthcare systems with rising healthcare demands and increasing costs, threatening the sustainability of national welfare systems. Somewhat surprisingly, the debate on costs of healthcare systems occur separately from another key challenge in healthcare: quality improvement and patient safety. In the aftermath of the publication of the now famous ‘To Err is Human’ report from the Institute of Medicine at the turn of the century (1999), healthcare organizations, professional bodies and healthcare regulators have attempted to improve quality of care through safety regulations (ie, procedures for medical errors and near incidents), standardizing care and – more recently – developing all sorts of initiatives to foster personalized healthcare delivery. While there is an abundance of literature within fields like Health Technology Assessment on the cost-effectiveness of interventions, quality instruments like guidelines are often developed without taking costst into account^1^ and even instruments which are meant to be integrated, like value-based healthcare, often focus on quality alone.^2^ The separation of ‘costs’ and ‘quality of care’ in organization or system-wide policies is surprising, however, in the light of contemporary debates on healthcare sustainability. Costs and quality have only incidentally been brought together in public outcries of healthcare leaders that the best care for individual clients is no longer possible. Such outcries underscore the urgency of the policy problem, stressing the need to studying the issues of costs and quality in tandem^[1]^.

 Wackers et al^3^ have made a valuable contribution to this emerging debate by bringing the issues of costs and quality together at the level of healthcare organisations. In their scoping review, they seek to provide overarching lessons by determining factors that influence the implementation of quality and cost policies in hospitals. From the literature, they identify 19 case studies that have succeeded in lowering costs whilst improving quality of care. This rather limited number of studies found illustrates the lack of scholarly attention for the topic of combining quality and costs at the organizational level. Their focus on organizations instead of procedures and organizational models (eg, Lean, Six Sigma) is interesting. It fits with the increasing recognition within social science that quality improvement is situated rather than universal, and that it must be ‘made’ into a good practice instead of ‘being implemented’ due to its political contestation, historical contingency and interpretive flexibility.^4^ As a consequence, good medical practices can never be homogenized into ‘best practices’ that can be scaled up, as quality initiatives always work out differently and must be adapted to local ways of reasoning and meaning-making that are often highly institutionalized.^5^ This is especially true for organization-wide initiatives such as studied by Wackers et al as these should be adapted to local circumstances, specificities of health systems, occupational roles, etc.

 This situated approach of quality and costs stresses the importance of narrative accounts (‘why does what work and how in this context’) and theoretical explanations that are able to carry a ‘good experience or result’ from one place to the next. In an often-cited study on infection prevention at intensive-care departments, Dixon-Woods et al^6^ argue for ‘ex-post theories’ that help to explain a quality phenomenon. They state that improvement programs almost never proceed as planned, and that the assumption that guide them and shape their actions usually change over time. Program leaders should not only be aware of this, but must also be able to anticipate and respond, using and contributing to theory building. This goal of theorizing is also pursued by Wackers et al who compose a list of 11 factors (ie, strategy, leadership, engagement, reorganization, finances, data and information, technology, project support, skill development, culture, communication) out of the selected literature that help to improve quality while reducing or containing costs. They for example stress the importance of supportive leadership that acknowledges the input from work floor levels, and the creation of a shared purpose and vision for engaging in organisation-wide change processes.

 Whilst their study is based on case studies, their paper remains rather abstract in the sense that they distract rather general ‘factors’ from the literature they studied. In the discussion, they themselves already state that these factors in themselves are not completely new. Moreover, by removing context, they run the risk that the factors themselves become a sort of checklist rather than a complex social intervention.^7^ Some of their factors are moreover in themselves complex social interventions; having a well running electronic patient record in place that supports organisation-wide monitoring for example is highly complex^8^ and one wonders how organisations with less well-developed information systems might still engage in organisation-wide improvement. For example, in a recent study on triage systems in older person care, training of care personnel and translating triage to the daily work of nursing aids were necessary to enable a rudimentary information system supporting task differentiation.^9^ Bringing in context thus is necessary to give substance to the factors distilled by Wackers et al.

 Such approaches would also allow for more insight in how ‘factors’ are accomplished in practice and how they work out in different settings. Much of the implementation literature uses a ‘factor generating’ approach that gives little insight into how such factors can be translated to specific settings and contexts. This is exactly what Dixon-Woods et al warned against in their *Explaining Michigan* paper referred to above. For this reason, several authors call for a more theory-informed approach to implementation.^10,11^ Such theoretical work could for example focus on the *work* needed for, or the *mechanisms* associated with quality improvement and cost containment. Such theoretical understandings can then also help to understand the interrelation between the factors. For example, in a study on integrated care for patients suffering from multimorbidity, attention is called for the *alignment work* performed by practitioners (healthcare professionals and managers alike) to connect the different elements that help develop, implement and sustain such programs.^12^

 Quality and cost issues increasingly transgress organizational boundaries, even more so these days with the pressing issue of workforce shortage that further threatens healthcare sustainability and accessibility. Solutions are found in inter-organizational collaboration, involving an increasingly diverse set of actors, including hospitals, nursing homes, rehabilitation clinics, family doctors, community nurses, etc. In many countries, for instance, experiments are held with processes of regionalisation of care, including new ways of allocating care, sharing information and spreading scarce capacities across organisational boundaries.^9,13,14^ Most of these studies are currently focused on the implementation of regional collaborative efforts, or at effects on accessibility of care. Following Wackers et al the effects on quality and costs of care should also be taken into account. This however requires new socio-economic evaluation models that address and connect social, policy, medical and economic issues, valuing ‘the broader picture.’ Further integration of such perspectives and the development of evaluation methodologies to capture the effects of such collaborative efforts thus seems warranted.

## Ethical issues

 Not applicable.

## Competing interests

 Authors declare that they have no competing interests.

## Authors’ contributions

 RB and IW made a set-up of the paper; IW wrote a first draft and RB finalized.

## Endnotes

 [1] Note, however, that some definitions of quality, like the one from the IoM, do implicitly include costs, by mentioning efficiency as part of quality. More common understandings, however, would refer to effectiveness, safety and patient-centredness, sometimes including timeliness and accessibility. In the Wackers et al paper, no explicit definition of quality is given, but we assume they also do not include costs.
